# Effect of increasing β-mannanase supplementation in diets containing copra meal on growth performance, meat quality, liver health, intestinal morphology, and nutrient utilization in broiler chickens

**DOI:** 10.5713/ab.24.0301

**Published:** 2024-08-26

**Authors:** Eun Cheol Lee, Kang Hyeon Kim, Min Sung Kang, Deok Yun Kim, Charline Mugeniwayesu, Dong Yong Kil

**Affiliations:** 1 Department of Animal Science and Technology, Chung-Ang University, Anseong 17546, Korea

**Keywords:** β-Mannanase, Broiler Chicken, Copra Meal, Energy and Nutrient Utilization, Growth Performance

## Abstract

**Objective:**

The current study aimed to investigate the effect of increasing β-mannanase supplementation in diets containing copra meal (CM) on growth performance, meat quality, liver health, intestinal morphology, and nutrient utilization in broiler chickens.

**Methods:**

A total of 1,600 3-d-old Ross 308 broiler chickens (initial body weight±standard deviation = 43.3±1.08 g) were randomly allotted to 1 of 5 treatment groups with 8 replicates. One group was fed a corn-soybean meal-based diet (control). Other 4 diets were prepared by inclusion of 10% commercial CM in the control diet with 0, 400, 800, and 1,600 U β-mannanase/kg. Experiments lasted for 32 d.

**Results:**

Birds fed the control diet had less (p = 0.001) feed conversion ratio (FCR) than those fed diets containing 10% CM without β-mannanase supplementation. Increasing supplementation of β-mannanase in diets containing 10% CM had no linear and quadratic effects on body weight gain, feed intake, and FCR in broiler chickens. The control diet had greater (p<0.01) apparent total tract retention (ATTR) of dry matter (DM), gross energy (GE), and N as compared to the diets containing 10% CM without β-mannanase supplementation; however, no differences in the ATTR of Ca and P were identified between 2 diets. There were no linear and quadratic effects of increasing supplementation of β-mannanase on the ATTR of DM, GE, N, Ca, and P in broiler diets containing 10% CM. Both inclusion of 10% CM and increasing supplementation of β-mannanase in broiler diets did not affect apparent metabolizable energy (AME) and N-corrected AME (AME_n_) values in treatment diets.

**Conclusion:**

The use of 10% CM in broiler diets during growing and finishing period impairs growth performance by decreasing energy and nutrient utilization in diets. Increasing β-mannanase supplementation in diets containing 10% CM has no positive effects on performance, meat quality, liver health, intestinal morphology, and nutrient utilization in broiler chickens.

## INTRODUCTION

The copra meal (CM) is a by-product of coconut oil production and the use of CM as an alternative feed ingredient in poultry feeds is steadily increased in many Asian countries because of increasing production of CM and its relatively cheap price [1]. However, feeding diets containing high amounts of CM to poultry may induce nutritional problems due to its poor essential amino acid profiles and high concentrations of non-starch polysaccharides (NSP; [2]). Previous studies reported that inclusion of 10% and 20% CM in grower and finisher broiler diets, respectively, impaired body weight gain (BWG) and feed conversion ratio (FCR) in broiler chickens [1,3]. However, Mael et al [1] reported that inclusion of up to 10% CM in both grower and finisher broiler diets maintained similar broiler performance as compared to the control diet. It is suggested, therefore, that dietary supplementation of NSP-degrading enzymes (NSPase) may promote the use of CM in broiler diets because of their capacity to break down antinutritional soluble NSP, such as β-mannan and galactomannan high in CM, which may realize increasing inclusion levels of CM in broiler diets [1,3]. However, little information regarding the response of broiler chickens to feeding diets containing high amounts of CM with supplementation of NSPase is currently available.

Dietary β-mannanase is a NSPase to target β-mannan chains of glucomannans and galactomannans in diets [4]. Therefore, dietary supplementation of β-mannanase is frequently applied in monogastric animal diets that contain feed ingredients high in β-mannan, such as palm kernel meal (PKM) and CM [5]. Previous experiments reported that dietary supplementation of β-mannanase in diets containing PKM or CM improved productive performance and health in pigs [6,7]. However, few experiments were performed to investigate the effect of dietary supplementation of β-mannanase in diets containing CM on growth performance, meat quality, health, and nutrient utilization in broiler chickens, although the efficacy of dietary enzyme complex including β-mannanase in diets containing CM was previously reported [1,3]. Furthermore, it can be hypothesized that increasing supplementation of β-mannanase in broiler diets containing CM by exceeding its conventional supplementation level may extend the beneficial effect because of the expectation to neutralize more antinutritional β-mannan in CM. However, this hypothesis of super-dosing effects of β-mannanase in broiler diets has not been tested before.

Therefore, the current study aimed to investigate the effect of increasing supplementation of β-mannanase in diets containing CM on growth performance, meat quality, liver health, intestinal morphology, and nutrient utilization in broiler chickens.

## MATERIALS AND METHODS

### Animal care

All experimental procedures were reviewed and approved by the Institutional Animal Care and the Use Committee (IACUC) at Chung-Ang University (Approval number: 2023 01020042).

### Experiment 1: growth trial

#### Animals, diets, and experimental design

A total of 1,600 3-d-old Ross 308 male and female broiler chickens (initial body weight [BW]±standard deviation = 43.3±1.08 g) were used and raised in a conventional floor pen. All chicks were randomly allotted to one of five dietary treatments with eight replicates per treatment. Each replicate consisted of 40 chicks. A two-phase feeding program with a grower diet from 3 to 21 d and a finisher diet from 22 to 35 d was used in this study. Within each phase, a corn-soybean meal-based control diet was formulated to meet or exceed the nutrient and energy concentrations as recommended in the Ross 308 manual [8]. One additional diet was prepared by inclusion of 10% commercial CM in the control diet with a partial replacement of corn and soybean meal. The nutritional compositions in CM used in this study are presented in Table 1. The inclusion level (i.e., 10%) of CM in treatment diets of this study was determined based on the previous experiment reporting the upper limits of inclusion levels of CM in diets for growing and finishing broiler chickens [1]. Diet compositions in 2 treatment diets for each phase are presented in Table 2. Three more treatment diets were produced by supplementation of 0.05%, 0.10%, or 0.20% β-mannanase (CTCzyme; declared activity of 800,000 U/kg; CTCBIO, Inc., Seoul, Korea) to diets containing 10% CM in replace of celite, leading to in-feed activities of 400, 800, and 1,600 U β-mannanase per kg diets in those 3 treatment diets, respectively. All treatment diets were prepared in a mash form.

Diets and water were provided to birds for 32 d of feeding trial from 3 to 35 d of age. The room temperature was maintained at 30°C during the first wk and then gradually decreased to 20°C at the end of the experiment. The average relative humidity was 58%±15% during the experiment. The experiment was conducted under a 24-h lighting scheme. The floor pens (200×230 cm; width×length for each pen) were furnished with fresh rice hulls for bedding materials. The BWG and feed intake (FI) were recorded at the conclusion of the experiment. Mortality was recorded daily. Following the correction for mortality, FCR was calculated by dividing FI by BWG [9].

#### Sample collection and analyses

At the conclusion of the experiment (i.e., 35 d of age), 1 male broiler chicken per replicate with a BW close to the replicate mean BW (i.e., 8 birds per treatment) was selected and euthanized by CO_2_ asphyxiation. The blood sample was immediately collected from each bird via a heat puncture into a 10-mL ethylenediaminetetraacetic acid tube (Becton and Dickinson company, Diagnostics, Berkshire, UK). This male bird was also used for the analyses of meat quality, liver health indicators, jejunal morphology, and digesta viscosity.

The breast muscle was excised to measure meat quality and stored at 4°C until further analyses. The right portion of breast meat was cut into four parts, two of which were used to analyze pH at 1 h and 24 h postmortem using a pH meter (Hanna Instruments, Nusfalau, Romanina), and the meat color was measured using a colorimeter, based on the Commission Internationale de l’Eclairage (CIE) color scale (Minolta Chroma Meter CR-400, Osaka, Japan) for lightness (L*), redness (a*), and yellowness (b*). The remaining two meats were used to measure water holding capacity (WHC) at 24-h postmortem and thiobarbituric acid-reactive substance (TBARS) value after storage at 4°C during 7 d, according to the methods described by Kim et al [10].

The collected whole blood samples were immediately centrifuged at 3,000×*g* at 4°C for 20 min to obtain the plasma. The supernatant was obtained in a 1.7-mL microtube (Axygen, Union City, CA, USA) and then stored in the refrigerator at −20°C before analyses. The plasma concentrations of alanine aminotransferase (ALT) and aspartate aminotransferase (AST) as the liver health indicator were analyzed using a Hitachi Automatic Analyzer 7020 (Hitachi Ltd., Tokyo, Japan). The subjective liver color score was also measured based on a scale from 1 to 5 (1 = dark red; 5 = yellowish red) to investigate the fatty liver incidence [11].

Jejunal morphology was examined by the method of Nari et al [12]. In short, 10% buffered formalin was used to flush and fix the sample. The jejunum fragment was embedded in paraffin. A 5-mm section of each paraffin-embedded sample was placed onto a glass slide, stained with hematoxylin-eosin, and examined under a light microscope. Villus height (VH), crypt depth (CD), villus width (VW), and VH to CD ratio (VH:CD) were determined with 20 measurements per jejunal sample. The detailed method was reported in the previous experiment [13].

The digesta samples in the part of the jejunum were collected and immediately stored at −20°C before analyses. Digesta viscosity was determined according to the method of Choct and Annison [14] with minor modification. Briefly, digesta samples were centrifuged at 9,000×*g* at 4°C for 10 min to obtain the supernatant. The relative viscosity was measured using an Ostwald viscosimeter with a ‘U’ shape tube containing two bulbs, two marks, and a capillary bore in one arm. The centrifuged digesta sample was drawn into the upper bulb of the viscosimeter by suction, allowing it to flow down through the capillary into the lower bulb. Two marks (one above and one below the upper bulb) indicated a known volume. The time required for the level of the fluid to pass between these marks was used for the values of the kinematic viscosity. The time for an aliquot of the digesta supernatant and distilled water to flow through the viscosimeter was recorded. The relative viscosity of a sample was calculated by dividing the former by the latter. This analysis was performed at the BT research facility center, Chung-Ang University.

### Experiment 2: metabolism trial

#### Animals, diets, and experimental design

A total of forty 36-d-old Ross 308 broiler chickens were allotted to 1 of 5 dietary treatments with 8 replicates per treatment and 1 bird per replicate. All birds were selected from the growth trial and were fed the same finisher diets used in the growth trial. Birds were placed in metabolic cages (35.2×45.0×55.3 cm; width×length×height). Room temperature was set at 21°C and the light was provided for 24 h throughout the experiment. The detailed experimental procedure was reported in our previous study [15].

#### Sample collection, chemical analyses, and calculation

The excreta were collected daily and immediately stored at −20°C. The excreta samples were dried in a force-air drying oven at 60°C for 48 h and finely ground for further analyses. The diets and excreta samples were analyzed for dry matter (DM; Method 934.01; [16]), nitrogen (N; Method 984.13; [16]), and gross energy (GE) using bomb calorimetry (Model 6400; Parr Instruments Co., Moline, IL, USA). The concentrations of calcium (Ca) and phosphorus (P) in the diets and excreta were also analyzed using inductively coupled plasma spectrometer (Optima 5300 DV; Perkin Elmer Inc., Shelton, CT, USA) as demonstrated by AOAC (Method 984.27; [16]) with minor modifications [17].

Apparent total tract retention (ATTR) of DM, GE, N, Ca, and P in treatment diets were calculated based on the previous method [18]. The values for apparent metabolizable energy (AME) and N-corrected AME (AME_n_) were also calculated with determined values for ATTR of GE and N [9,19].

### Statistical analyses

All data for both growth and metabolism trials were analyzed in a completely randomized design using PROC MIXED procedure of SAS (SAS Institute., Cary, NC, USA). Each replicate was considered an experimental unit for all analyses. The outlier data were checked by the UNIVARIATE procedure of SAS; however, no outliers were found. The LSMEANS procedure was used to calculate the treatment means. The PDIFF option was used to compare treatment means if the difference in treatment means was significant. In addition, non-orthogonal polynomial contrast tests were performed to investigate the linear and quadratic effects of increasing supplementation levels of β-mannanase in diets containing 10% CM without the data for the control diet [20]. A probability of p<0.05 was considered significant.

## RESULTS

### Growth performance and meat quality

Birds fed the control diet had less (p = 0.001) FCR than those fed diets containing 10% CM without β-mannanase supplementation, whereas no differences in BWG and FI were found between 2 treatments (Table 3). Increasing supplementation of β-mannanase up to 0.20% in diets containing 10% CM had no linear and quadratic effects on BWG, FI, and FCR in broiler chickens. However, supplementation of 0.05% β-mannanase in broiler diets containing 10% CM had less (p<0.05) FCR than supplementation of 0.10% β-mannanase.

All meat quality measurements including pH, meat color, WHC, and TBARS were not affected by both inclusion of 10% CM and increasing supplementation of β-mannanase in broiler diets containing 10% CM (Table 3). However, polynomial contrast tests revealed breast meat color with the linear increase in L* value (p = 0.049) but the linear decrease in a* value (p = 0.028) by increasing supplementation of β-mannanase in broiler diets containing 10% CM. Nevertheless, no such a linear and quadratic effect was observed in other meat quality measurements.

### Liver health indicator

Birds fed the control diet showed no differences in plasma AST and ALT as well as liver subjective color score compared with birds fed diets containing 10% CM without β-mannanase supplementation (Table 4). Likewise, increasing supplementation of β-mannanase up to 0.20% in broiler diets containing 10% CM had no linear and quadratic effects on all liver health indicators.

### Jejunal morphology and digesta viscosity

Jejunal morphology including VH, VW, CD, and VH:CD were not influenced by both inclusion of 10% CM and increasing supplementation of β-mannanase in broiler diets containing 10% CM (Table 5). Similarly, no effects of dietary treatments on jejunal digesta viscosity were observed in this experiment.

### Energy and nutrient utilization in diets

The control diet had greater (p<0.01) ATTR of DM, GE, and N as compared to the diets containing 10% CM without β-mannanase supplementation; however, no differences in ATTR of Ca and P were identified between 2 broiler diets (Table 6). There were no linear and quadratic effects of increasing supplementation of β-mannanase on ATTR of DM, GE, N, Ca, and P in broiler diets containing 10% CM. However, the broiler diet containing 10% CM with 0.20% β-mannanase supplementation had greater (p<0.05) ATTR of N than the broiler diet containing 10% CM with 0.10% β-mannanase supplementation. Both inclusion of 10% CM and increasing supplementation of β-mannanase in broiler diets containing 10% CM did not affect AME and AME_n_ values for treatment diets.

## DISCUSSION

Copra meal is widely used as an alternative ingredient in poultry diets because of its relatively high amounts of energy and nutrients as well as low prices despite poor nutritional values such as high fiber and poor amino acid profiles [2]. It was reported that inclusion of 10% CM in broiler diets decreased feed costs but had no negative effects on productive performance in broiler chickens, further suggesting that the supplementation of dietary NSPase may promote the inclusion levels of CM at more than 10% in broiler diets [1]. This result was one of the reasons why we decided to include 10% CM in treatment diets of this study and hypothesized that increasing supplementation levels of β-mannanase (i.e., β-mannanase activity) may further improve nutritional values of diets containing 10% CM. However, we found that feeding diets containing 10% CM to broiler chickens increased FCR as compared to feeding the control diet despite no effects on meat quality, intestinal morphology, digesta viscosity, and liver health. This observation may be associated with decreased energy and nutrient utilization in diets because the current study also showed decreased ATTR of DM, GE, and N in broiler diets by inclusion of 10% CM. The reason for decreased energy and nutrient utilization by inclusion of CM in broiler diets has been related to its high concentrations of fiber and physiochemical properties limiting the efficient digestion and absorption in the intestinal tract [2,21]. Therefore, different from the previous finding [1], our results may indicate that inclusion of 10% CM in broiler diets has an adverse impact on growth performance in broiler chickens by lowering energy and nutrient utilizations.

Copra meal is known to contain high amounts of soluble NSP, especially in the type of galactomannan and mannan [21,22]. Therefore, dietary β-mannanase may be considered a primary choice as a supplemental enzyme in diets containing CM because β-mannanase can specifically target and break down β-mannan in diets, leading to an expectation of mitigating its antinutritional effects of β-mannan [5]. However, to our knowledge, there have been very few experiments regarding the response of broiler chickens to β-mannanase supplementation in diets containing CM. Sundu et al [23] reported that supplementation of enzyme complex including β-mannanase in diets containing CM improved growth performance and nutrient utilization in broiler chickens. However, Mael et al [1] reported that dietary supplementation of enzyme complex including β-mannanase had no positive effects on productive performance in broiler chickens fed diets containing 10% CM. Since there is a lack of information for the effect of β-mannanase supplementation in broiler diets containing CM, we hypothesized that increasing supplementation of β-mannanase in broiler diets containing CM may improve productive performance, health, and nutrient utilization in broiler chickens possibly with a linear manner because CM contains high amounts of β-mannan and more β-mannanase supplementation neutralizes more antinutritional β-mannan. However, this hypothesis failed to be proven because of no linear or quadratic effects of increasing supplementation of β-mannanase in diets containing 10% CM. The reason for this result is difficult to explain due to the limited previous data in poultry; however, the relatively small amounts of β-mannan in CM may be one reason because dietary β-mannanase supplementation was reported to improve productive performance in broiler chickens when PKM, which contains more β-mannan than CM, was included in broiler diets [5,24]. Moreover, unexpectedly high amounts of available energy in diets containing 10% CM may be another reason. In this experiment, we measured AME_n_ values for finisher diets containing 10% CM in the metabolism trial and the measured values were greater by 45 to 89 kcal/kg than the originally calculated AME_n_ values (i.e., 3,200 kcal/kg). This high amount of available energy in treatment diets containing CM may mask beneficial effects of increasing supplementation of dietary β-mannanase in broiler chickens.

In the current experiment, we also investigated the effect of increasing supplementation of β-mannanase in diets containing 10% CM on meat quality, liver health indicators, intestinal morphology, digesta viscosity, and nutrient utilization in broiler chickens; however, its linear and quadratic effects on most measurements were not significant. Interestingly, a linear increase in L* value but a linear decrease in a* value for breast meat color were observed by increasing supplementation of β-mannanase in diets containing 10% CM. However, the differences appeared to be very small among treatments and the measured values were closed to typical values observed in the breast meat color of broiler chickens [22,25], which indicated that increasing supplementation of β-mannanase in diets containing 10% CM had little impact on breast meat color in broiler chickens as observed in other measurements of the current study. However, it should be noted that, to our best knowledge, this experiment is the first to investigate various physiological responses of broiler chickens to increasing β-mannananse supplementation (i.e., up to 1,600 U/kg) in broiler diets containing high amounts of CM. More research regarding interactive effects of different inclusion levels of CM and β-mannananse supplementation in broiler diets is required.

## CONCLUSION

The use of 10% CM in broiler diets during growing and finishing period impairs growth performance in broiler chickens by decreasing energy and nutrient utilization in diets. However, increasing supplementation of up to 0.20% β-mannanase (i.e., 1,600 U/kg) in broiler diets containing 10% CM has no positive impact on growth performance, meat quality, liver health, intestinal morphology, digesta viscosity, and utilization of energy and nutrients in broiler chickens.

## Figures and Tables

**Table 1:** Analyzed nutrient concentrations of the copra meal used in this experiment (as-fed basis)

Items	Values
Dry matter (%)	90.5
Gross energy (kcal/kg)	4,021
Crude protein (%)	21.2
Neutral detergent fiber (%)	48.4
Acid detergent fiber (%)	33.1
Ether extract (%)	2.2
Total amino acids (%)	
Trp	0.14
Arg	1.84
His	0.34
Ile	0.61
Leu	1.25
Lys	0.41
Met	0.31
Phe	0.81
Thr	0.62
Val	0.91
Ala	0.85
Asp	1.56
Cys	0.42
Glu	3.60
Gly	0.86
Pro	0.68
Ser	0.85
Tyr	0.35

**Table 2:** Composition and nutrient concentrations of experimental diets

Items	Grower phase		Finisher phase	
	
	Control	Copra meal	Control	Copra meal
Ingredients (%)				
Corn	55.18	45.33	59.79	51.85
Soybean meal, 45% CP	27.80	23.61	22.75	15.00
Corn gluten meal	7.53	8.16	6.48	9.32
Copra meal	-	10.00	-	10.00
Tallow	3.95	7.19	6.08	8.66
Monodicalcium phosphate	1.78	1.77	1.52	1.52
Limestone	1.45	1.46	1.26	1.30
L-Lysine HCl (99%)	0.49	0.59	0.40	0.59
DL-Methionine (98%)	0.32	0.34	0.27	0.27
L-Threonine (99%)	0.19	0.22	0.14	0.17
L-Tryptophan (98%)	0.03	0.03	0.02	0.03
Celite	0.30	0.30	0.30	0.30
Salt	0.20	0.20	0.20	0.20
50% Choline	0.10	0.10	0.10	0.10
NaHCO_3_	0.20	0.20	0.20	0.20
Coccidiostat	0.10	0.10	0.10	0.10
Antioxidant	0.20	0.20	0.20	0.20
Vitamin premix^1)^	0.10	0.10	0.10	0.10
Mineral premix^2)^	0.10	0.10	0.10	0.10
Total	100.00	100.00	100.00	100.00
Energy and nutrient content^3)^				
AME_n_ (kcal/kg)	3,050	3,050	3,200	3,200
Crude protein (%)	22.25	22.25	19.50	19.50
Digestible Lys (%)	1.22	1.22	1.03	1.03
Digestible Met (%)	0.64	0.65	0.56	0.56
Digestible Met+Cys (%)	0.91	0.91	0.80	0.80
Digestible Thr (%)	0.82	0.82	0.69	0.69
Digestible Trp (%)	0.19	0.19	0.16	0.16
Total calcium (%)	0.92	0.92	0.79	0.79
Available phosphorus (%)	0.46	0.46	0.40	0.40

1) Provided per kg of the complete diet: vitamin A, 12,000 IU (retinyl acetate); vitamin D_3_, 4,000 IU; vitamin E, 80.0 mg; vitamin K_3_, 4.0 mg (menadione dimethylpyrimidinol); vitamin B_1_, 4.0 mg; vitamin B_2_, 10.0 mg; vitamin B_6_, 6.0 mg; vitamin B_12_, 20.0 μg; folic acid, 2.0 mg; biotin, 200 μg; niacin, 60 mg.

2) Provided per kg of the complete diet: iron, 60 mg (FeSO_4_); zinc, 100 mg (ZnSO_4_); manganese, 120 mg (MnO); copper, 16 mg (CuSO_4_); cobalt, 1,000 μg (CoSO_4_); selenium, 300 μg (Na_2_SeO_3_); iodine, 1.25 mg [Ca(IO_3_)_2_].

3) Calculated values from CVB [26].

**Table 3:** Effect of β-mannanase supplementation in diets containing copra meal on growth performance and breast meat quality in broiler chickens^1)^

Items		Dietary treatments					SEM	p-value^2)^		

		CON	Supplemental levels of β-mannanase in 10% CM diets							
	
			0%	0.05%	0.10%	0.20%		Total	Linear	Quadratic
Growth performance										
BWG (kg)		2.06	2.03	1.99	1.97	2.02	0.028	0.175	0.885	0.112
FI (kg)		3.15	3.19	3.12	3.16	3.20	0.092	0.422	0.452	0.158
FCR (kg/kg)		1.53^c^	1.57^ab^	1.57^b^	1.60^a^	1.59^ab^	0.033	0.001	0.201	0.350
Breast meat quality										
pH	1 h	6.08	6.01	6.00	6.14	6.13	0.101	0.208	0.060	0.550
	24 h	5.64	5.75	5.71	5.74	5.76	0.068	0.301	0.686	0.644
Meat color	L*	45.3	43.0	44.1	45.2	44.9	0.81	0.102	0.049	0.140
	a*	3.49	4.39	4.36	3.11	3.11	0.510	0.136	0.028	0.503
	b*	12.1	12.5	12.3	12.9	13.4	0.66	0.650	0.268	0.785
WHC (%)		70.5	73.8	69.5	68.7	70.9	1.87	0.384	0.410	0.070
TBARS		0.45	0.57	0.46	0.46	0.48	0.043	0.290	0.248	0.117

CON, corn and SBM-based control diets; CM, copra meal; SEM, standard error of means; BWG, body weight gain; FI, feed intake; FCR, feed conversion ratio; L*, lightness; a*, redness; b*, yellowness; WHC, water holding capacity; TBARS, thiobarbituric acid-reactive substances.

1) Data are least squares means of 8 observations per treatment.

2) Total = overall effects of treatments; Linear and quadratic effects of increasing β-mannanase supplementation in 10% CM diets without the data for CON treatment.

a–c Means with different superscripts within a row differ (p<0.05).

**Table 4:** Effect of β-mannanase supplementation in diets containing copra meal on liver health indicators in broiler chickens^1)^

Items	Dietary treatments					SEM	p-value^2)^		

	CON	Supplemental levels of β-mannanase in 10% CM diets							
	
		0%	0.05%	0.10%	0.20%		Total	Linear	Quadratic
Plasma AST (U/L)	309	279	311	274	297	22.9	0.615	0.810	0.970
Plasma ALT (U/L)	1.73	1.83	1.71	1.91	1.56	0.259	0.640	0.327	0.445
Liver color score	1.11	1.15	1.23	1.18	1.23	0.090	0.844	0.638	0.899

CON, corn and SBM-based control diets; CM, copra meal; SEM, standard error of means; AST, aspartate aminotransferase; ALT, alanine aminotransferase.

1) Data are least squares means of 8 observations per treatment.

2) Total = overall effects of treatments; Linear and quadratic effects of increasing β-mannanase supplementation in 10% CM diets without the data for CON treatment.

**Table 5:** Effect of β-mannanase supplementation in diets containing copra meal on jejunal morphology and digesta viscosity in broiler chickens^1)^

Items	Dietary treatments					SEM	p-value^2)^		

	CON	Supplemental levels of β-mannanase in 10% CM diets							
	
		0%	0.05%	0.10%	0.20%		Total	Linear	Quadratic
VH	1,152	1,196	1,288	1,200	1,219	63.6	0.661	0.935	0.721
VW	130	130	126	134	132	6.0	0.936	0.658	0.889
CD	153	150	174	142	148	16.2	0.710	0.624	0.855
VH:CD	9.0	9.3	9.7	9.8	9.7	0.87	0.962	0.771	0.727
Digesta viscosity, cPs	1.78	1.70	1.74	1.70	1.71	0.044	0.632	0.941	0.791

CON, corn and SBM-based control diets; CM, copra meal; SEM, standard error of means; VH, villus height; VW, villus width; CD, crypt depth.

1) Data are least squares means of 8 observations per treatment.

2) Total = overall effects of treatments; Linear and quadratic effects of increasing β-mannanase supplementation in 10% CM diets without the data for CON treatment.

**Table 6:** Effect of β-mannanase supplementation in diets containing copra meal on energy and nutrient utilization in broiler chickens^1)^

Items	Dietary treatments					SEM	p-value^2)^		

	CON	Supplemental levels of β-mannanase in 10% CM diets							
	
		0%	0.05%	0.10%	0.20%		Total	Linear	Quadratic
Energy values (kcal/kg)									
AME	3,379	3,395	3,438	3,387	3,420	36.0	0.148	0.655	0.881
AME_n_	3,217	3,249	3,289	3,245	3,266	34.7	0.389	0.827	0.955
ATTR of nutrients (%)									
DM	76.6^a^	73.0^b^	72.9^b^	72.6^b^	73.9^b^	0.54	<0.001	0.233	0.232
GE	80.2^a^	77.5^b^	77.4^b^	77.2^b^	78.0^b^	0.56	0.001	0.386	0.348
N	62.3^a^	55.9^bc^	57.4^bc^	54.5^c^	59.2^ab^	1.38	0.004	0.174	0.211
Ca	35.5	30.1	32.9	27.5	32.6	3.19	0.352	0.730	0.532
P	37.7	33.4	34.8	34.1	37.4	2.02	0.478	0.206	0.728

CON, corn and SBM-based control diets; CM, copra meal; SEM, standard error of means; AME, apparent metabolizable energy; AME_n_, nitrogen-corrected apparent metabolizable energy; ATTR, apparent total tract retention; DM, dry matter; GE, gross energy; N, nitrogen; Ca, calcium; P, phosphorus.

1) Data are least squares means of 8 observations per treatment.

2) Total = overall effects of treatments; Linear and quadratic effects of increasing β-mannanase supplementation in 10% CM diets without the data for CON treatment.

a–c Means with different superscripts within a row differ (p<0.05).
